# Identification of the Midgut Microbiota of *An. stephensi* and *An. maculipennis* for Their Application as a Paratransgenic Tool against Malaria

**DOI:** 10.1371/journal.pone.0028484

**Published:** 2011-12-06

**Authors:** Navid Dinparast Djadid, Hoda Jazayeri, Abbasali Raz, Guido Favia, Ignacio Ricci, Sedigheh Zakeri

**Affiliations:** 1 Malaria and Vector Research Group (MVRG), Biotechnology Research Center (BRC), Pasteur Institute of Iran, Tehran, Iran; 2 School of Biosciences & Biotechnology, University of Camerino, Camerino, Italy; French National Centre for Scientific Research - Université Aix-Marseille, France

## Abstract

The midgut microbiota associated with *Anopheles stephensi* and *Anopheles maculipennis* (Diptera: Culicidae) was investigated for development of a paratransgenesis-based approach to control malaria transmission in Eastern Mediterranean Region (EMR). Here, we present the results of a polymerase chain reaction (PCR) and biochemical-based approaches to identify the female adult and larvae mosquitoe microbiota of these two major malaria vectors, originated from South Eastern and North of Iran. Plating the mosquito midgut contents from lab-reared and field-collected *Anopheles* spp. was used for microbiota isolation. The Gram-negative and Gram-positive bacterial colonies were identified by Gram staining and specific mediums. Selected colonies were identified by differential biochemical tests and 16S rRNA gene sequence analysis. A number of 10 *An. stephensi* and 32 *An. maculipennis* adult mosquitoes and 15 *An. stephensi* and 7 *An. maculipennis* larvae were analyzed and 13 sequences of 16S rRNA gene bacterial species were retrieved, that were categorized in 3 classes and 8 families. The majority of the identified bacteria were belonged to the *γ-proteobacteria* class, including *Pseudomonas sp.* and *Aeromonas sp.* and the others were some closely related to those found in other vector mosquitoes, including *Pantoea, Acinetobacter, Brevundimonas, Bacillus, Sphingomonas, Lysinibacillus and Rahnella*. The 16S rRNA sequences in the current study aligned with the reference strains available in GenBank were used for construction of the phylogenetic tree that revealed the relatedness among the bacteria identified. The presented data strongly encourage further investigations, to verify the potential role of the detected bacteria for the malaria control in Iran and neighboring countries.

## Introduction

Malaria, tuberculosis and AIDS are the most important infectious diseases in the world, while malaria has been considered as the major parasitic and vector-borne disease in Iran and also the main health problem in South-Eastern parts of the country. Across the Kerman, Hormozgan and Sistan and Baluchestan provinces, around 10,000 cases of malaria infections have been reported in 2008 and 4.1 million people are considered to be at risk of infection (Centers for Disease Control and prevention (CDC), Iran, unpublished data).

Since the discovery that mosquitoes are the vectors of malaria parasite by Ronald Ross [1], [2], vector control has become an important part of malaria control programs. Several strategies have been designed and put into place to reduce the mosquito population, and several others are currently being investigated as possible solutions for rendering the mosquito vector less competent to transmit malaria. These strategies include environmental management, insecticide treatments and molecular entomology approaches [3]. One of the newly developed approaches that has been proposed as an anti-Plasmodium effector delivery strategy is paratransgenesis, which is the genetic modification of symbiotic microorganisms to deliver anti-pathogenic products and thus reduce vector competence[4].

Paratransgenesis studies were started by Durvasula *et al.* [5] to break the transmission of American trypanosomiasis (*Trypanosoma cruzi*) by *Rhodnius prolixus*, which, in turn, fostered attempts for controlling other diseases like malaria. In early 1960s, a few studies were carried out on midgut microbiota of laboratory-bred species of *Culex* [6], [7], [8]. Further, the presence of oxidase-positive bacteria from the midgut of anopheline mosquitoes [9] and also, the successful colonization of *Serratia marcescens* in laboratory-bred *An. stephensi* were reported [10]. A study of wild *Aedes triseriatus*, *Cx. pipiens*, and *Psorophora columbiae* using routine laboratory bacteriologic techniques indicated the presence of *S. marcescens*, *Klebsiella ozonae*, *Pseudomonas aeruginosa*, and *Enterobacter agglomerans* [11]. Moreover, the examination of the *Cx. quinquefasciatus* larvae midgut indicated the presence of bacteria represented by *Bacillus* spp., *Staphylococcus* spp. and *Pseudomonas* spp., while *Aspergillus* and *Streptomyces* spp. represented the fungal and actinomycete inhabitants, respectively. Chao and Wistreich [7], Vasanthi and Hoti [12], Jadin *et al.* [9] found *Pseudomonas* sp. in the midgut of mosquitoes from the Democratic Republic of the Congo. Straif *et al.* [13] identified 20 different genera of midgut bacteria from *An. gambiae* sensu lato and *An. funestus* mosquitoes caught in Kenya and Mali. They identified *Pantoea agglomerans* (synonym *Enterobacter agglomerans*) as the most frequently isolated bacterium, apart from *Escherichia coli*. Gonzalez-Ceron *et al.* [14] isolated *Enterobacter amnigenus*, *Enterobacter cloacae*, *Enterobacter* sp., *Serratia marcescens*, and *Serratia* sp. from *An. albimanus* mosquitoes caught in southern Mexico.

Pidiyar *et al.* [15] reported the isolation and taxonomic characterization of some species, *Aeromonas culicicola*, from the midgut of *Cx. quinquefasciatus* and two strains of *A. culicicola* from *Aedes aegypti*, indicating that different mosquito species in the same environment may harbor common representatives of the microbiota. Pidiyar *et al.* [16] studied the midgut microbiota of wild *Cx. quinquefasciatus* and Lindh *et al.* [17] reported 16 bacteria species from 14 genera in *An. gambiae* sensu lato and *An. funestus*.

In this report, we studied the midgut microbiota of Iranian *An. stephensi* and *An. maculipennis* mosquitoes by using biochemical identification and molecular techniques with the aim to identify suitable candidates to develop an effective strategy for malaria control in Iran and perhaps Eastern Mediterranean region.

## Results and Discussion

Here, we have presented the results of culture-dependent biochemical tests and polymerase chain reaction (PCR)-based approach to identify midgut bacteria associated with *An. stephensi* and *An. maculipennis*, two major malaria vectors in Iran.

Plating the mosquito midgut contents from lab-reared and field-collected *An. stephensi* and *An. maculipennis* (Female adult/larvae) and also the water of their breeding sites was used for isolation of culturable microflora. At first, prepared samples were cultured on blood Agar medium and next, different morphological colonies (based on the colony size, shape, color and margin) were cultured on Blood Agar (for isolation of Gram-positive bacteria) and MacCONKEY media (for isolation of Gram-negative bacteria) in parallel.

The Gram-negative bacteria colonies were identified by culture-dependent biochemical tests such as IMViC, oxidase and etc. Also, the Gram-positive bacteria colonies were studied by Gram staining (for morphology characteristics) and supplemental tests such as Bacitracine sensitivity, catalase, coagulase, Novobiocine sensitivity, Optochine and etc. They were further selected on the basis of colony characteristics too. The selected colonies were identified by the analysis of the 16S rRNA gene sequence.

From 10 *An.stephensi* and 32 *An. maculipennis* adult mosquitoes and 15 *An. stephensi* and 7 *An. maculipennis* larvae analyzed, we retrieved 13 sequences of 16S rRNA gene bacterial species in 3 classes and 8 families.

**Figure 1:**
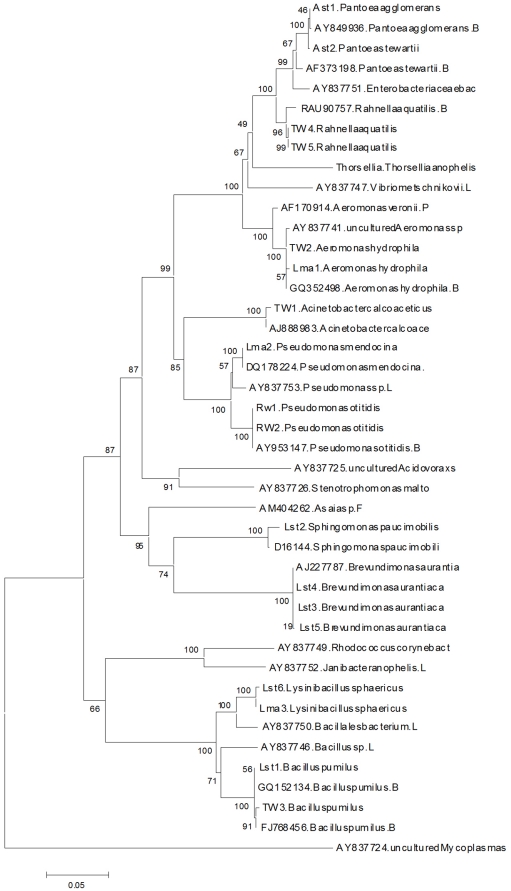
Phylogenetic dendrogram constructed in Mega4 based on 16S rRNA gene sequences. Bacteria species released in GenBank are appeared with accession number and species of bacteria. Those bacteria detected in current study are Ast (Adult sample of *An. stephensi*), Lst (Larvae of *An. stephensi*), Lma (Larvae of *An. maculipennis*), RW (Rasht breeding sites), TW (Talesh breeding sites).

The majority of 16S rRNA sequences detected are from bacteria belonging to the *Gammaproteobacteria* class (Table 1). The *Pseudomonas sp.* and *Aeromonas sp.* are species detected in most of the specimens analyzed by two biochemical and molecular pathways. These species are found in breeding sites of both *An. stephensi and An. maculipennis* in South and North-eastern of Iran and in *Anopheles* larvae and adult specimens (Tables 2 and 3). However, although there is no significance co-relation among the bacterial species and anopheles species or specific breeding site, but for example, *Lysinibacillus sphaericus* have been detected in both larvae of *An. maculipennis* and *An. stephensi* that originated from two different zoogeographical regions of Palearctic and Oriental, or *An. stephensi* larvae share two bacteria of *Bacillus pumilus* and *Rahnella aquatilis* with the Talesh breeding site in north of country (Tables 2 and 3). This result is encouraging because the use of paratransgenesis requires a bacterium that could be introduced to the majority of the mosquito vector populations and could be transferred within breeding sites to different stages of larvae, pupae and adults. Many of the 16S rRNA sequences retrieved from our samples are similar to those of gut bacteria found in other mosquitoes. The *Aeromonas* and *Pseudomonas* species were isolated from *Cx. quinquefasciatus* [15] in India, *An. gambiae* [17] in Africa, *An. darlingi* in Brazil [18] and field collected *An. stephensi* larvae [19] in India. Some of our sequences deduced from breeding sites and *Anopheles* larvae (Lma2, RW1, RW2 strains) are closely related to the *Pseudomonas sp.* clone H2.26 (AY837753) from adult *An. gambiae* [17] (Fig. 1).

**Table 1:** Details of molecular identification of bacteria species in *An. stephensi, An. maculipennis* specimens and breeding places.

NO.	Strain	Nearest species	Ac. no. of identified bacteria	Maxidentity	Ac. no. of the closest speciesin gene bank	Class	Family	Product size(bp)
1	Ast1L	*Pantoea agglomerans*		99	AY849936.1	Gammaproteobacteria	Enterobacteriaceae	1500
2	Ast2L	*Pantoea stewartii*		98	AF373198.1	Gammaproteobacteria	Enterobacteriaceae	911
3	Lst1L	*Bacillus pumilus*		99	GQ152134.1	Bacillales	Bacillaceae	1507
4	Lst2L	*Sphingomonaspaucimobilis*		98	D16144.1	Alphaproteobacteria	Sphingomonadaceae	1447
5	Lst3F	*Brevundimonasaurantiaca*		99	AJ227787.1	Alphaproteobacteria	Caulobacteraceae	1419
6	Lst4F	*Brevundimonasaurantiaca*		99	AJ227787.1	Alphaproteobacteria	Caulobacteraceae	1419
7	Lst5F	*Brevundimonasaurantiaca*		99	AJ227787.1	Alphaproteobacteria	Caulobacteraceae	1419
8	Lst6F	*Lysinibacillus sphaericus*		99	CP000817	Bacillales	Planococcaceae	1510
9	Lst7F	*Rahnella aquatilis*		98	U90757.1	Gammaproteobacteria	Enterobacteriaceae	1501
10	Lma1F	*Aeromonas bivalvium*		99	DQ504429.1	Gammaproteobacteria	Aeromonadaceae	951
11	Lma2F	*Pseudomonasmendocina*		100	DQ178224.1	Gammaproteobacteria	Pseudomonadaceae	1495
12	Lma3F	*Lysinibacillus sphaericus*		99	CP000817.1	Bacillales	Planococcaceae	1510
13	Lma4F	*Aeromonas punctata*		95	GQ259885.2	Gammaproteobacteria	Aeromonadaceae	1446
14	RW1	*Pseudomonas otitidis*		99	AY953147.1	Gammaproteobacteria	Pseudomonadaceae	1495
15	RW2	*Pseudomonas otitidis*		99	AY953147	Gammaproteobacteria	Pseudomonadaceae	1496
16	TW1	*Acinetobactercalcoaceticus*		99	AJ888983.1	Gammaproteobacteria	Moraxellaceae	1508
17	TW2	*Aeromonas hydrophila*		99	GQ184148.1	Gammaproteobacteria	Aeromonadaceae	1506
18	TW3	*Bacillus pumilus*		99	FJ768456.1	Bacillales	Bacillaceae	1507
19	TW4	*Rahnella aquatilis*		98	U90757.1	Gammaproteobacteria	Enterobacteriaceae	1500
20	TW5	*Rahnella aquatilis*		98	U90757.1	Gammaproteobacteria	Enterobacteriaceae	1500

Ast (Adult sample of *An. stephensi*), Lst (Larvae of *An. stephensi*), Lma (Larvae of *An. maculipennis*), RW (Rasht Breeding Sites), TW: (Talesh Breeding Sites). L and F are lab-reared and field-caught specimens, respectively.

**Table 2:** Molecular identification of bacteria species based on breeding sites, adults and larvae of mosquitoes.

Sourceof sample		Identified bacteria species				
**Breeding sites**	**Talesh**	*Rahnella aquatilis*	*Aeromonas hydrophila*	*Bacillus pumilus*	*Acinetobacter calcoaceticus*	
	**Rasht**	*Pseudomonas otitidis*				
**Larvae sample**	***An. maculipennis***	*Pseudomonas mendocina*	*Aeromonas punctata*	*Aeromonas bivalvium*	*Lysinibacillus sphaericus*	
	***An. stephensi***	*Bacillus pumilus*	*Brevundimonasaurantiaca*	*Lysinibacillussphaericus*	*Sphingomonaspaucimobilis*	*Rahnella aquatilis*
**Adult sample**	***An. stephensi***	*Pantoea agglomerans*	*Pantoea stewartii*			

**Table 3:** Molecular identification of bacteria species based on common bacterial species in each source.

Bacteria species		Pan. agg	Pan. ste	Bac. pum	Sph. pau	Bre. aur	Lys. sph	Rah. aqu	Aer. biv	Pse. men	Aer. pun	Pse. oti	Aci. cal	Aer. hyd
Sources of samples		1	2	3	4	5	6	7	8	9	10	11	12	13
**Breeding sites**	**Talesh**			•				•					•	•
	**Rasht**											•		
**Larvae sample**	***An. maculipennis***						•		•	•	•			
	***An. stephensi***				•	•	•							
**Adult sample**	***An. stephensi***	•	•	•										

• 1(Pantoea agglomerans), 2(Pantoea stewartii), 3(Bacillus pumilus), 4 (Sphingomonas paucimobilis), 5(Brevundimonas aurantiaca), 6(Lysinibacillus sphaericus), 7(Rahnella aquatilis), 8(Aeromonas bivalvium), 9(Pseudomonas mendocina), 10(Aeromonas punctata), 11(Pseudomonas otitidis), 12(Acinetobacter calcoaceticus), 13(Aeromonas hydrophila).

Other detected bacteria species in the current study, including *Pantoea, Bacillus* and *Acinetobacter* (Tables 1, 2, and 3), have been reported recently from different Culicidae mosquitoes. *Pantoea* sp. was found in *Cx. quinquefasciatus* [15] in Africa and *An. darlingi* in Brazil [18]. Fouda *et al.* concluded that the isolated *Bacillus* from a laboratory colony of *Culex pipiens* mosquitoe midguts were essential for high and normal fecundity [20]. *Bacillus sp.* was also reported from African *An. gambiae* [17], *Cx. quinquefasciatus* [15] and *An. stephensi* larvae in India [19]. *Acinetobacter sp.* was found in *Cx. quinquefasciatus* [15] and field-collected male, female and larvae of *An. stephensi* [19].

Besides, some other bacteria species, including *Acinetobacter bomani*, *Pseudomonas florsen* and *Enterobacter sp.* from breeding sites, *Pseodomonas aerogenosis* from adult *Anopheles*, and *Acinetobacter, E. coli, Klebsieila ozenae* and *Shigella sp.* from *Anopheles* larvae, were identified just by using the biochemical pathway. These species were also identified from some mosquitoes by molecular pathway. Lindh *et al.* [17] and Rani *et al.* [19] reported *Escherichia sp.* from *An. arabiensis* and field-collected male *An. stephensi*, respectively.

Terenius *et al.* [18] reported 56 sequences of 16S rRNA gene of 56 bacterial species from six mosquitoes, including five blood-fed and one non blood-fed mosquitoes. They extracted DNA from whole mosquitoes, which could be one of the reasons for detection of a large number of bacteria, whereas some bacteria from cuticle or other unrelated parts of mosquito's body can be reported in spite of their irrelevance to paratransgenic approach. Although Terenius *et al.* [18] assumed that non blood-fed mosquitoes did not amplify any product, indicating that bacteria on the exoskeleton of the mosquitoes were too few to affect the results; our results do not confirm this whereas most samples in our current study were non-blood-fed. The other reason for large numbers of bacteria in Terenius *et al.* [18] study, could be using the blood-fed samples because the bacterial load increases after blood meal.

Similarly to our result, Lindh *et al.* [17] detected bacteria in a low percent of the mosquitoes that had been analyzed using the same PCR conditions. Some of the identified bacteria (*Pseudomonas, Aeromonas, Bacillus*) in breeding sites were also detected in larvae (molecular identification) and adults (biochemical identification) *(Pseudomonas)*, but according to low number of identified samples in adult it should be examined further. Although we had enough adult mosquitoes, from 32 examined blood-fed and non blood-fed adult *An. maculipennis* we could not find any bacteria whereas in all *An. stephensi* mosquitoes, the bacteria detection was successful.

In the current study, specimens were identified by biochemical and culture-dependent pathways. Moreover, in spite of using two different pathways, including culture-independent and culture dependent in some recent papers, we did not use culture-independent pathway because by using culture independent pathway (PCR based methods), the identified genera could not be retrieved by the culture-dependent method [17]. One explanation might be the remnants of the midgut cells or human blood interfere in PCR reaction. Another explanation could be the competition of DNAs from different bacteria that led to the successful amplification of ones with the higher abundance [17].

So far, several bacteria have been isolated from anophelines, from both laboratory colonies and wild populations and at least one bacterium, *Enterobacter agglomerans*, has been identified as an excellent candidate for paratransgenesis [13], [21]. Inhibition of malaria parasite development has been shown in Yoshida *et al.* [22] study where *E. coli* was fed to *An. stephensi*.Using a single-chain antibody against *Plasmodium berghei* ookinete, Pbs21 linked to the lytic peptide Shiva-1, resulted in 95.6% transmission blockage. Furthermore, in a recent study, the expression of two anti-*Plasmodium* effector molecules (SM1 and phospholipase-A2 (PLA2)) on the surface of *E. coli*, partially inhibited the *P. berghei* development, showed the potential role of this strategy for malaria control programs [21], [23].

The data presented from the current study revealed a great diversity of the midgut microbiota in *An. stephensi* and *An. maculipennis* and presented potentially new species. This justifies further search and isolation of useful bacteria for paratransgenesis.

All of the bacterial isolates in this study will be further evaluated for their suitability as paratransgenic tools. The first step will be the sustainability study in mosquito midguts after reintroduction and the second step is the assessment of the immune response that induced by the bacteria. The survival of a reintroduced bacterium will depend on the tolerance level to immune response that mounted by the mosquito and putative antagonistic effects of other midgut bacteria. Several studies have shown that Gram-negative midgut bacteria can suppress *Plasmodium* parasites development [14], [24], [25], possibly by acting as elicitors of the mosquito immune response that affecting on *Plasmodium* development [25]. Hence, an ideal bacterium for paratransgenics would be one that elicits a potent immune response that suppresses other bacteria and *Plasmodium* parasites but does not affect on own survival. Therefore, genetic modification of this bacterium by introducing a gene that expresses an antiparasitic molecule, could achieve the clearance of *Plasmodium* parasites from the mosquito midgut. From this point of view, it is promising that several isolates detected in the current study are Gram-negative, proteobacteria, and these are suitable elements for genetic modification [17].

Further, recently a great deal of attention has been addressed to bacteria of the genus *Asaia*, which have been proposed as the best potential candidates for malaria control [26], [27], [28]. In the original screening, we could not identify *Asaia* that this is most likely due to the fact that it grows in very peculiar conditions [26]. Also, bacterial identification by molecular analysis can be strongly biased by the kind of primers that are used in ribosomal gene amplification. However, by using *Asaia* specific primers, we could not detect its presence in any of the analyzed samples.

On the other hand, Cirimotich et al [29]identified an Enterobacter bacterium isolated from wild mosquito populations in Zambia that renders the mosquito resistant to infection with the human malaria parasite *Plasmodium falciparum* by interfering with parasite development before invasion of the midgut epithelium. These means that despite the use of identified bacteria in paratransgenic strategy, it is also possible to raise the anti-Plasmodium mechanism through mediating a mosquito-independent interaction of small populations of replicating bacteria with the malaria parasite, which is reported to be largely caused by bacterial generation of reactive oxygen species.

Accordingly, the detection of 13 sequences of 16S rRNA gene bacterial species (categorized in 8 families) in two main malaria vector species, *An. maculipennis* from Palearctic region and *An. stephensi* from western extend of Oriental region in current study, strongly encourage further investigations, to verify the potential role of the detected bacteria in malaria control in Iran and neighboring countries.

## Materials and Methods

### Ethics Statement

All projects, prior to the approval by Pasteur institute of Iran (PII) and Ministry Of Health (MOH), should had been reviewed and cleared by the ethical committee of PII and hence, in mosquito collection from private residences, all residents provided their oral informed consent to have their residences used in the study by coordination through “Local Manager of Malaria Control Program” of Public Health Center in the study areas. Briefly, the PI for the project, N. Dinparast Djadid (NDD), presented the different sections of project to the local staff, especially the field collection section. One to two weeks prior to field collection, the “Local Manager of Malaria Control Program” of Public Health Center, had a visit from the study areas and in a meeting with residents, asked their permission for mosquito collection in the defined date and time. As it is a routine procedure for other activities of malaria control program in the region, the residents accepted to cooperate in the project, which followed by the presence of the research team in the agreed date and time within the study areas and performing the mosquito collection. This has been documented in “Weekly Activity Report Book” of the Malaria Control Program in the study areas, signed by the PI (NDD), the manager of malaria control program and a representative from the study areas.

### Collection of mosquito species and isolation of bacterial flora

**Figure 2:**
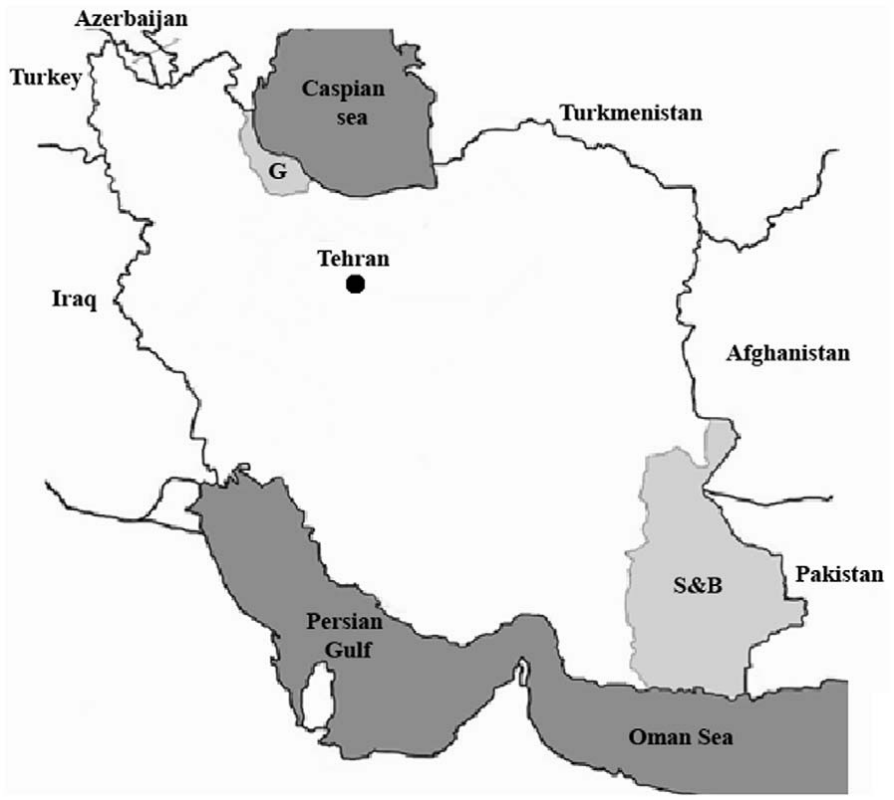
Map of study area, including Guilan (G), Sistan and Baluchestan (S&B) provinces.

Adult and larvae mosquito specimens of *An. stephensi* and *An. maculipennis* were collected by total catch and hand catch in human and animal shelters and also their breeding sites from provinces of Sistan and Baluchestan in South West of Iran and Guilan in northern Iran (Fig. 2). Specimens species were identified by using the morphological key of Iranian Anophelines [30] to distinguish *An. stephensi* and *An. maculipennis* adults from other *Anopheles* species. The details of the origin and the number of specimens that were sequenced and the detected bacteria have been presented in Table 1.

Living mosquitoes were anesthetized by putting them on ice and dissection was done under sterile conditions after surface sterilization with 70% ethanol. The midgut content was suspended in 50 µl of sterile saline (0.9% NaCl); A 25 µL aliquot of this content was plated onto blood agar base (Merck, Germany) with 7% (v/v) human blood, and 25 µL aliquot of the content was plated onto MacConkey agar base (Merck, Germany) followed by incubation at 37°C for 18–24 hours. The sterility of all reagents was checked during the entire procedure. The morphology of the Gram-positive bacteria was examined by Gram staining and light microscopy. IMVic, oxidase and different types of supplemental analysis were used for classical phenotyping, depending on the bacterial genus.

Water of the breeding sites were collected in 50 ml sterile falcon tubes and transferred to local laboratories for further process. On arrival, the waters centrifuged in 10,000 rpm for 3 minutes and the pellet from each water was plated on blood agar and MacConkey mediums, followed by biochemical and molecular identification of their bacteria, as carried out for the detection of microbiota of the mosquitoes' midgut.

### Genomic DNA extraction, amplification, cloning and sequencing of 16S rRNA genes

The optimum growth of bacteria was gained in LB by shaking at 160 rpm at 37°C and spectrophotometric reading. DNA was extracted by using the phenol- chloroform extraction protocol for Gram-negative and -positive bacteria according to the adapted protocols in Microbiology Department, Pasteur Institute of Iran.

The 1.5 kb of the 16 s rRNA gene was amplified by using 16 s forward (5′-AGT TTG ATC CTG GCT CAG-3′) and 16 s reverse (5′-GCT ACC TTG TTA CGA CTT C-3′) primers that were designed by Gene Runner,Version 3.05. All PCR reactions were performed in a total volume of 25 µl. The reaction mixture contained 100 ng of each of the specific primers, 0.2 unit of Taq polymerase, 0.1 mM each of dNTPs, 0.001% spermidin, 2.5 µl of 10× reaction buffer and 2 mM MgCl_2_. The amplification profile was as follows: denaturation at 95°C for one minute, followed by 35 cycles of annealing at 54°C for one minute and extension at 72°C for one minute with 10 minute extra extension time in the last cycle.

Specific forward and reverse primers were designed based on the 16 s rRNA gene of *Asaia* bacteria Gene Runner, Version 3.05. Asafor (5′-GCG CGT AGG CGG TTT ACA C-3′) and Asarev (5′-AGC GTC AGT AAT GAG CCA GGT T-3′) [26] were used to amplify a 180 bp fragment. The expected size of PCR products were confirmed by electrophoresis on a 1% agarose gel that was stained with ethidium bromide and the bands were visualized by UV transillumination. Amplified fragments were purified by Core-One™ Gel Elution Kit (Core Bio System Co. Ltd., Seoul, Korea) and PCR products with the expected size were cloned into pDrive TA cloning vectors (QIAGEN). The inserted gene in the plasmids was subjected to sequencing by Millegene Company (Labege, France), using M13 primers [(M13 (−21) and M13R (−29)].

### Data analysis and phylogenetic tree

The sequencing signals of the specimens were double-checked and annotated, followed by comparison with GenBank data. For data analysis, alignment and construction of phylogenetic tree, a series of softwares, including DNASTAR, Lasergene (Version 7.1), Chromas (Version 2.31), Gene Runner (Version 3.05), ClustalX (Version 1.83), ClustalW (1994), Blast (http://www.ncbi.nlm.nih.gov/BLAST/) and other programs that were available in NCBI site and Malaria and Vector Research Group (MVRG), were used.

To determine the phylogenetic relatedness of the strains, the 16S rRNA gene sequence of the mosquito midgut contents were subjected to analysis by using Molecular Evolutionary Genetic Analysis (MEGA4, 2007) software.
