# Correction: Identification of the Midgut Microbiota of *An. stephensi* and *An. maculipennis* for Their Application as a Paratransgenic Tool against Malaria

**DOI:** 10.1371/annotation/8d703875-d295-420f-aa9e-a26988876929

**Published:** 2012-01-04

**Authors:** Navid Dinparast Djadid, Hoda Jazayeri, Abbasali Raz, Guido Favia, Irene Ricci, Sedigheh Zakeri

There is an error in the given name of the fifth author. The correct name is Irene Ricci.

